# Epigenetic mechanisms in cardiovascular complications of diabetes: towards future therapies

**DOI:** 10.1186/s10020-024-00939-z

**Published:** 2024-09-27

**Authors:** Giulia Damiano, Raffaella Rinaldi, Angela Raucci, Chiara Molinari, Annalisa Sforza, Sergio Pirola, Francesco Paneni, Stefano Genovese, Giulio Pompilio, Maria Cristina Vinci

**Affiliations:** 1 Unit of Vascular Biology and Regenerative Medicine, Centro Cardiologico Monzino IRCCS, Via C. Parea 4, Milano, 20138 Italy; 2 Unit of Cardiovascular Aging, Centro Cardiologico Monzino IRCCS, Milano, 20138 Italy; 3 Diabetes, Endocrine and Metabolic Diseases Unit, Centro Cardiologico Monzino IRCCS, Milano, 20138 Italy; 4 Department of Cardiac Surgery, Centro Cardiologico Monzino IRCCS, Milan, Italy; 5 Center for Translational and Experimental Cardiology (CTEC), Department of Cardiology, University Hospital Zurich and University of Zürich, Zürich, Switzerland; 6 University Heart Center, University Hospital Zurich, Zurich, Switzerland; 7 Dipartimento di Scienze Biomediche, Chirurgiche e Odontoiatriche, Università degli Studi di Milano, Milano, 20100 Italy

**Keywords:** Diabetes, Cardiovascular complications, Epigenetics, Epidrugs GLP-1RA

## Abstract

The pathophysiological mechanisms of cardiovascular disease and microvascular complications in diabetes have been extensively studied, but effective methods of prevention and treatment are still lacking. In recent years, DNA methylation, histone modifications, and non-coding RNAs have arisen as possible mechanisms involved in the development, maintenance, and progression of micro- and macro-vascular complications of diabetes. Epigenetic changes have the characteristic of being heritable or deletable. For this reason, they are now being studied as a therapeutic target for the treatment of diabetes and the prevention or for slowing down its complications, aiming to alleviate the personal and social burden of the disease.

This review addresses current knowledge of the pathophysiological links between diabetes and cardiovascular complications, focusing on the role of epigenetic modifications, including DNA methylation and histone modifications. In addition, although the treatment of complications of diabetes with “epidrugs” is still far from being a reality and faces several challenges, we present the most promising molecules and approaches in this field.

## Introduction

Diabetes mellitus (DM) is a chronic metabolic disease due to absence of insulin secretion or to insulin resistance and impaired pancreatic islet secretion combined in many different phenotypes. Hyperglycaemia, the hallmark of both type 1 and type 2 DM, is the main initiating factor in the pathogenesis of diabetic complications. Considering that globally almost 346 million people suffer from diabetes and it is predicted that by 2050 will affect almost one to three people, vascular complications will exert a huge burden in terms of death and disability, quality of life and healthcare costs (Lu et al. 2023). Despite the recognition and implementation of recent guidelines suggesting tight glycaemic control, the appearance and progression of macrovascular and microvascular diseases have been only partially affected.

DM and impaired glucose tolerance increase cardiovascular disease (CVD) risk 3- to 8-fold (Giacco and Brownlee 2010) whereas microvascular complications are still the leading cause of morbidity, and mortality, and reduced life expectancy by 5–15 years in patients with diabetes. This underlies the need to better understand the mechanisms underpinning DM vascular complications, which are known to persist and progress despite glucose lowering therapies and metabolic risk factors optimization. The purpose of this review is to present an update of most recent explorative avenues for the treatment of diabetes and its complications.

## Diabetes complications

DM is associated with both macrovascular and microvascular lesions (Liu et al. 2022). These vascular complications are wide-ranging and are in part result in chronic exposure of blood glucose. The macrovascular complications are due to damage to large arteries, leading to myocardial, limb or cerebrovascular ischemia events (Li et al. 2023). Differently, microvascular complications, i.e. retinopathy, nephropathy and neuropathy, are due to damage of small blood vessels, causing important morbidity and mortality. In addition to “classic” diabetes complications, thanks to longer life expectancy and increased clinical awareness, recent studies have shown a significant link between DM and liver fibrosis, cognitive decline, functional disability, affective disorders, obstructive sleep apnoea, and cancer (Tomic et al. 2022).

However, although most of the DM complications are well known in their clinical developement, the underlying mechanisms are still unclear.

## Biochemical mechanisms in diabetic complications

Several studies have shown that oxidative stress is a key element in the development and progression of DM-associated complications (Rains and Jain 2011). At the cellular level, DM-induced metabolic abnormalities are known to promote mitochondrial superoxide production (Giacco and Brownlee 2010). This main upstream event is responsible for the final activation of numerous pathways involved in hyperglycaemia-induced cellular/tissue damage.

Increased polyol pathways flux, hexosamine synthesis pathway, pentose phosphate pathways, and intracellular advanced glycation end-product (AGEs) production deriving from the non-enzymatic reaction between glucose with proteins or lipids, are some of the glycolytic metabolic pathways activated by the excessive intracellular concentration of glucose and involved in ROS production (Semba et al. 2010; Perrone et al. 2020) in DM, as already extensively and recently reviewed by Chen et al. (Chen et al. 2023). The ROS molecules can mediate cellular toxicity by reacting with proteins (especially cysteine residues), lipids (lipid peroxidation), nucleic acids (DNA damage and strand breaks), as well as signaling pathways such as the NFkB transcription factor (Nakajima and Kitamura 2013), and diacylglycerol (DAG)-protein kinase C (PKC) pathway, causing pathological changes in gene expression. Interestingly, elevated glucose levels can directly activate the DAG-PKC pathway by increasing de novo synthesis of the lipid messenger DAG, an activator cofactor of the PCK family (Noh and King 2007). The hyperactivation of PKC induced by hyperglycaemia is responsible for numerous deleterious effects at the level of different tissues such as reduced expression of the endothelial nitric oxide (NO) synthase (eNOS) and increased expression of vasoconstrictor endothelin-1 in endothelium, or increased production of extracellular cell matrix (ECM) and TGF-β1 expression in mesangial cells or renal glomeruli responsible for blood flow abnormalities and diabetic nephrotic syndrome respectively (Noh and King 2007; Geraldes and King 2010).

## Metabolic memory

Once hyperglycaemia develops vasculopathies appear and progress in DM patients. However, patients who achieve an early tighter glycaemic control benefit more than others in terms of event- and complication-free survival, even years after adopting a looser glucose management. Likewise, having poor control in early years after diabetes onset is associated to higher rate of diabetes complications, even after restoration and maintenance of normoglycemia with drugs, diet and/or exercise.

This suggests that cells can record the prior glucose exposure and perpetuate an altered metabolic profile, even after normoglycemia is restored. This phenomenon, termed “metabolic memory” or “legacy effect”, has been extensively observed and described in last 40 years on microvascular complications development, both in type 1(The relationship of 1995; Epidemiology of Diabetes I and Complications Research 1999) and type 2 diabetes (Cencioni et al. 2014; Waddington and CANALIZATION OF DEVELOPMENT AND THE INHERITANCE OF ACQUIRED CHARACTERS 1942; Tronick and Hunter 2016; Dupont et al. 2009) and led to recent guidelines recommendations, that suggest to obtain and maintain tighter glucose control from diabetes onset and onward to confer long-lasting protective effects against complications (Giacco and Brownlee 2010; Lachin et al. 2021; Nathan et al. 2005).

Although the effects on the development of microvascular complications are clear and well described, the available data on a possible legacy effect on macrovascular complications do not allow a definitive conclusion on the existence of a long-term protective effect of tight control on major adverse cardiovascular events (MACE). Results suffer from the finding of increased mortality in the intensive glycaemic control group in the ACCORD study. These results are most likely affected by the use, during those trials, of drugs with safety and cardioprotection profiles different from those available today, in particular considering risk for hypoglycaemic events. Nevertheless, a recent systematic review (Prattichizzo et al. 2020) reports that intensified glucose control was associated with significantly decreased risk of cardiovascular events with higher effect on individuals with shorter duration of diabetes (< 10 years) and no pre-existing cardiovascular disease. A review of observational studies also suggests some evidence for a legacy effect in real-life populations. The observation that duration of diabetes has a modifying effect on metabolic memory suggests that mechanisms underlying micro- and macrovascular complications, once triggered due to hyperglycaemia, become (partly) irreversible. Consequently, achieving tight glycaemic control later in the natural history of diabetes could confer a less protective effect.

So far, metabolic memory represents an obstacle (and therefore a potential therapeutic target) in the treatment and management of DM and its complications, therefore the identification of the mechanisms underlying this phenomenon has been of great interest in the field of diabetes (Cencioni et al. 2014).

## The emerging role of epigenetics

“Epigenetics” (epi- “over, around”) means “above genetics”, outside the traditional Mendelian genetic basis for inheritance. This term was first coined by the British scientist C.H. Waddington in 1942 (Waddington and CANALIZATION OF DEVELOPMENT AND THE INHERITANCE OF ACQUIRED CHARACTERS 1942). Weddington thought of “epigenetics” as a model by which genetic components interact with their surroundings in response to environmental changes. He was referring exclusively to epigenetics in the field of cell differentiation and cell development. He depicted the totality of all possible combinations of adaptation as a hilly “epigenetic landscape” and the “decision” against a particular direction of cell fate as valleys and forks. Waddington asks the reader to imagine cell lineage commitment as some marbles rolling down the epigenetic landscape following the grooves, a mechanism called “canalization”(Tronick and Hunter 2016).

Today, epigenetics is defined as the study of mitotically and/or meiotically heritable changes in gene function that do not affect DNA sequence (Dupont et al. 2009). Environment, nutrition, lifestyle and drugs shape cells’ reaction and adaptation, and this phenotypic plasticity occurs thanks to epigenetic mechanisms (Ecker et al. 2018). Epigenetics act as a bridge between the fixed genome and phenotypic plasticity displayed by cells, creating more variability in the normal flow of the central dogma in genetics, wherein information flows in one only direction: DNA-RNA-proteins (Chang and Qi 2023). These modifications play a physiologically role during development, both in stem cells differentiation and in mature differentiated cells. In this way it is explained how a single DNA sequence can produce more than 400 different cell types (Breschi et al. 2017).

## Chromatin architecture

The cell nucleus of eukaryotic cells is a membrane-bound organelle composed of heterogeneous, dynamically changing biomolecules, of which a large fraction is represented by chromatin, a tight association of genomic DNA with histone proteins in which the nucleosome is the functional unit (Camara and Mascher 2023). This is composed of a segment of DNA (about 146 bp) wrapped in 1.67 left-handed turns around eight histone proteins. Each histone octamer encompasses two copies of H2A, H2B, H3 and H4. The histone H1, at the entry site of DNA, interacts with the segment of DNA linker between nucleosomes(Siebel et al. 2010). Histones are globular basic proteins enriched in arginine (Arg) and lysine (Lys) residues and their positive charge allow the formation of non-covalent bonds with the DNA phosphate groups. Core histones share a conserved domain of three α-eliches, while the N-terminal basic tail protrude from the nucleosome, leaving the amino acids undergo a great variety of posttranslational modifications (PTMs) that affects their interaction with DNA (Bajbouj et al. 2021).

Chromatin is subject to different types of PTM affecting the amino termini of histones, regulating access to the underlying DNA. Chromatin remodelling is responsible for the dynamic component of the fixed genotype and, from a functional and structural point of view, chromatin occurs in two higher-order structures: euchromatin and heterochromatin. The first is uncondensed, gene rich and thus transcriptionally active; the latter is compact, gene poor and inaccessible for the transcription machinery (Camara and Mascher 2023).

## Epigenetic control of gene expression

The major epigenetic changes constitute DNA methylation, histone modification and non-coding RNA. The first two modification close and open the chromatin structure to regulate the accessibility of the DNA from the transcriptional factors and are performed by specific enzymes that based on their function are named “writers” or “erasers” when add or remove specific marks. The non-coding RNA control the gene expression at the RNA level (Handy et al. 2011).

### DNA methylation

This epigenetic modification consists in the covalent addition of methyl groups (-CH3) to the fifth carbon of a cytosine ring, that is converted in methyl cytosine 5 (5mC). This modification is principally found in the CpG dinucleotide sequences, but recent findings suggest that the methylation of non-CpG cytosine is crucial for gene regulation in embryonic stem cells, suggesting that the methylation plays an important role in the maintenance of the pluripotent state (Lister et al. 2009). The methylation reaction is catalysed by DNA methyltransferases (DNMT) enzymes, through the transfer of -CH3 from an S-adenosyl-I-methionine (SAM) to the cytosine residue. There are several DNMT enzymes: DNMT3A and DNMT3B set up DNA methylation patterns during the early phase of development, working in complex with DNMT3L, a regulatory protein; DNMT1 is called the “maintenance” enzyme and it copies the existing methylation patterns during DNA replication, but it needs DNMT3A and DNMT3B to exploit its function (Jones 2012).

**Fig. 1:**
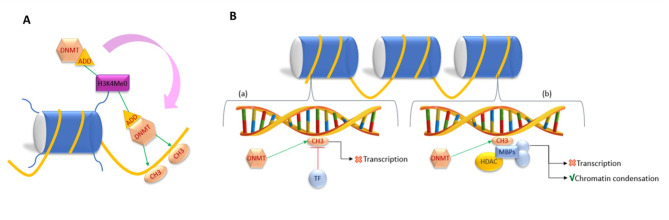
Mechanism of function of DNMT3A. (**A**) DNMT3A recognizes and bind histone H3 when methyl-free and this interaction causes a conformational change that led DNMT3A to an active state. Thus, DNMT3A is ready to catalyse DNA methylation. (**B**) Schematic representation of repression of transcriptional activity mediated by DNA methylation

It has been reported that DNA methylation is associated with specific histone methylation patterns: the presence of H3K9me3 and the absence of H3K4me (H3K4me0). De novo methylation is catalysed by DNMT3A and DNMT3B in complex with DNMT3L. These methyltransferases share together a domain called ADD that recognize methyl-free H3 tail. When DNMT3A recognizes H3K4me0 through its ADD domain, an allosteric modification of DNMT3A occurs and stimulates the methylation activity of DNMT3A catalytic domain on DNA molecule (Fig. 1A) (Torres and Fujimori 2015).

In mammals, the DNA molecules composing centromeres, telomeres and repetitive elements are highly methylated, thus heterochromatic and inactive. This pattern confers stability and suppresses for example the expression of transposable elements preventing genome rearrangements (Bhat et al. 2022). In contrast, CpG islands are generally methyl-free and function as promoters for approximately 60% of human genes (Ginno et al. 2012). Generally, methylation in a gene promoter exerts a repressive activity, while methylation at the level of gene body is positively correlated with gene expression. Enhancers, that can be very distant from promoters, are mostly CpG-poor and they have a dynamic pattern of methylation (Jones 2012).

The methylation can suppress transcription by several mechanism. First, the presence of a methyl group can directly block transcription factor (TF) DNA recognition and binding (Fig. 1B). Alternatively, methyl-CpG-binding proteins (MBPs) represented by MBD1, MBD2, MBD3, MBD4, and MeCP2 preferentially bind the methylated sites and recruit transcriptional co-repressor molecules to silence transcription. MBPs form complexes with histone deacetylases (HDAC) and corepressors, leading to chromatin condensation and inhibiting transcriptional activities (Fig. 1B) (Klose and Bird 2006).

Methylation patterns can be lost after replication either when the mechanism methylation maintenance fails or when demethylase enzymes erase the mark. The ten-eleven translocation (TET) family of proteins (TET1, TET2 and TET3) catalyses the oxidation of 5mC to 5-hydroxymethylcytosine (5hmC), and to other minor metabolites 5-formylcytosine (5fC) and 5-carboxylcytosine (5ac), making them able to erase the DNA methylation (Klose and Bird 2006).

### Histones post-translational modifications

The two most studied histone modifications are methylation and acetylation. In addition, histones residues can undergo phosphorylation, ubiquitination, SUMOylation, glycosylation, citrullination and many other modifications have been discovered recently (Impivaara et al. 1986; Cavalieri 2021).

These epigenetic marks lead to changes in chromatin conformation, that become more/less prone to the binding of transcriptional machinery. PTMs modulate chromatin structure by altering electrostatic interactions between histone proteins and DNA and by modifying the recruitment of various non-histone proteins that act as coactivators and corepressor to chromatin (Cavalieri 2021; Musselman et al. 2012).

Histone methylation occurs through the transfer of a methyl group (-CH3) to lysine (K) and arginine (A) residues on histones H3 and H4. Lysine methylation is the most versatile modifications: the residue can be mono, di or tri methylated. Only mono and demethylation have been described for arginine residues. While methylation of DNA is generally associated with gene silencing, histone methylation on lysine residue, catalysed by histone methyl transferase HMT, can lead both to activation and inhibition (Miller and Grant 2013). Methylated residue is identified and bounded by chromodomains-containing proteins that are involved both in the formation of inactive chromatin and transcriptional activation, depending on the position of the methylated amino acid: for example, H3K9, H3K27 and H4K20 are repressors, while the methylation of H3K4 and H3K36 is an activator signal (Yap and Zhou 2011). Methylation marks can be then reversed by lysine demethylases (Thinnes et al. 2014) a class of enzymes that in humans is represented by two distinct subfamilies of demethylases (KDMs), the flavin-dependent KDM1 subfamily and the 2-oxoglutarate- (2OG) dependent JmjC subfamily, both employing oxidative mechanisms (Thinnes et al. 2014).

Histones ‘tails are rich in lysine residues which are target for acetylation marks. Histone acetylation occurs through the transfer of an acetyl group (-COCH3) from acetyl coenzyme A, catalysed by histone acetyltransferase (HAT) enzymes. HATs activities are generally correlated with enhanced gene expression: acetylation neutralizes the charge of the histones reducing the affinity of histone’s tail for DNA, favouring chromatin relaxation (Eberharter and Becker 2002; Costantino et al. 2018). HATs are divided into two groups: type A and type B. Type A HATs are nuclear enzymes containing acetyl-CoA binding sites and are related to gene transcription whereas type B HATs play a role in the maintenance of cell functioning. As cytoplasmic enzymes they modify free histones after synthesis in the cytoplasm and transport them to the nucleus, where they are deacetylated and incorporated into chromatin. In addition, HATs can be divided into four families based on their primary structure: p300/CBP (p300 and CBP), MYST (Esa1, MOF, Sas2, Sas3, MORF, Tip60 and Hbo1), GNAT (Gcn5, PCAF, Hat1, Elp3 and Hpa2) and Rtt109 (Verza et al. 2020).

The enzymes involved in deacetylating lysine residues are the histone deacetylases (HDAC). They lead to chromatin compaction and repress gene expression. So far, 18 HDACs have been identified in humans, which are divided into four classes: class I (HDAC1-3, and HDAC8) which have nuclear localization. Class IIa (HDAC4-5, HDAC7, and HDAC9) and IIb (HDAC6 and HDAC10) characterized by tissue specific expression and can shuttle between the nucleus and cytoplasm, suggesting a role of this class in the acetylation of non-histone proteins. The Class III are Sir2-type proteins (SIRT1-7). The specific expression pattern of this class is unknown, and its mechanisms differ from those of the other two classes. The protein of class IV (HDAC11) shows homology to both class I and class II members (Verza et al. 2020; Wang et al. 2014). Both HATs and HDACs operate as catalytic subunits within major protein complexes. Acetylated lysine is recognized and bound by bromodomain-containing proteins that recruit proteins involved in transcription or chromatin decompaction (Haery et al. 2015). Among these proteins, bromodomain and extraterminal (BET) proteins bind acetylated histone marks and coordinate the recruitment of RNA Polymerase II. Their role will discuss later in this review.

### Non-coding RNAs

Around 98% of our genome is transcribed but not all RNAs are translated into proteins. These DNA products are termed non-coding RNAs (ncRNAs). Non-coding RNAs (ncRNAs) are ubiquitous and based on size are classified into 2 major groups: (i) small ncRNAs (sncRNAs, < 200 nucleotides long) including microRNAs, endogenous short interfering RNAs, PIWI-interacting RNAs, and (ii) long non-coding RNAs (lncRNAs), ranging from 200 nts and 2kb (Zhang et al. 2019). Both have been reported to have epigenetic effects with important functions in the regulation of cell differentiation, development cell cycle regulation and aging (Zhang et al. 2019). This area of research is relatively recent and there are many ongoing investigations about ncRNAs role in epigenetic control (Mattick et al. 2023).

## The histone code: writers, erasers and readers

Histone marks and DNA methylation are not alone able to regulate chromatin conformation. Other non-histone proteins operating in a larger chromatin context are also involved in the control of gene expression. Methylated DNA and DNA methyltransferases can promote the recruitment and assembly of regulatory proteins involved in the formation of condensed chromatin structures that are inaccessible to the transcriptional machinery. In this regard, DNA methyltransferases, have been proved to bind histone methyltransferase and histone deacetylase, thus propagating their signal towards a more complex chromatin structure (Klose and Bird 2006). MeCP2 is a methyl-CpG binding protein with 2 domains: a methyl-CpG binding domain (MBD) at the amino-terminal and a transcriptional repression domain (TRD) that interacts with deacetylase complex. So, there is a tight correlation between DNA methylation and deacetylation of the core histones, which result overall in gene silencing (El-Osta and Wolffe 2000). DNA and histone lysine methylation or acetylation are dynamic modification regulated by enzymes that introduce or reduce these epigenetic marks. The crosstalk between DNA and histone methylation is performed by proteins that bind histone marks to facilitate or inhibit the assembly of transcriptional machinery (readers). The variety of environmental stimuli and histone modifications, the synergistic or antagonistic effect of epigenetic marks, the number of enzymes involved and the crosstalk between DNA and histone modifications contribute to the complexity of the epigenetics field. Epigenetic “writers” establish specific pattern of signatures on histones’ tails that are recognized by non-histone proteins, called the “readers”, that regulate chromatin structure and function. These modifications can be reverse by the “eraser” molecules. This hypothesis is called “histone code”. Because of the close correlation between DNA methylation and histone modification, it is better referred to as the ‘epigenomic code’, indicating the combination of patterns of both DNA and histone modifications. Coordination between readers, writers and erasers plays a key role in the dynamics of the epigenetic code, regulating substrate specificity and recruitment, thus modulating DNA readout (Torres and Fujimori 2015).

## Epigenetic readers: BET-proteins

**Fig. 2:**
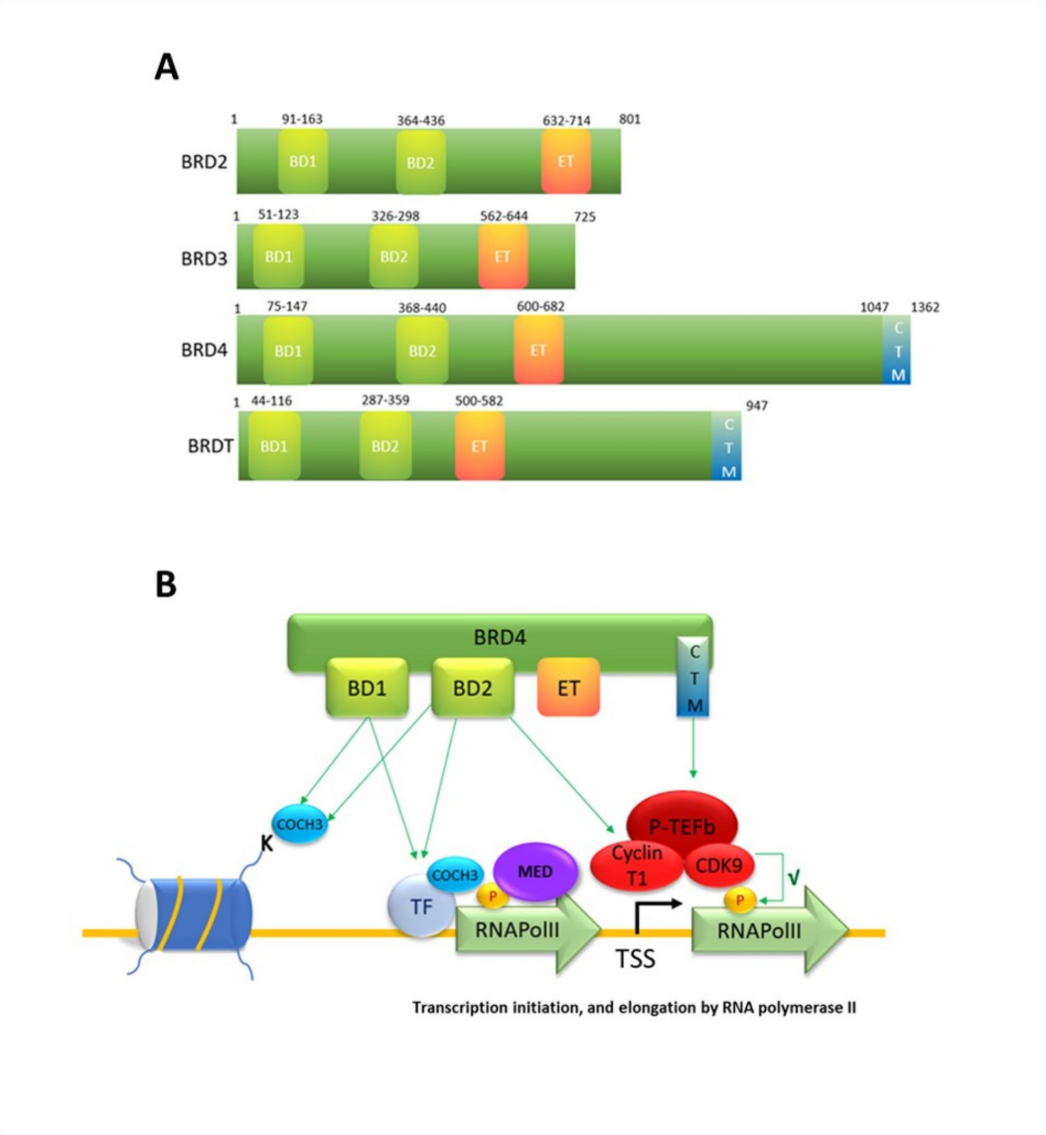
(**A**) Representative structure of BET proteins. (**B**) Functions of BET family member BRD4 in the regulation of promoter. BRD4 binds to acetylated lysine (COCH3) in histones or transcription factors (TF) via its BD1 and BD2 domains. The binding of acetylated histones by BRD4, at transcription start sites (TSS), mediates transcriptional co-activation and elongation via RNA polymerase II (RNA pol II) and Mediator (Med) and positive transcription elongation factor B (P-TEFb) signaling complexes

In the interplay between writers and erasers, there are reader proteins, able to recognize a specific modification and facilitate the assembly of the transcriptional machinery to drive gene expression. Among these readers, the bromodomain and extra-terminal (BET) domain-containing proteins. BET proteins family belongs to a superfamily of bromodomain-containing protein and comprises BRD2, BRD3 and BRD4 and the testis-specific isoform BRDT. All the components possess 2 bromodomains BD1 and BD2 and an extraterminal (ET) domain (Fig. 2A) that can bind the histone acetylation, facilitating transcription factors, RNA polymerase II (RNAPol-II) and coactivators recruitment to target gene promoters and enhancers (Guo et al. 2023; Ali et al. 2022; Borck et al. 2020).

The domains comprise a two tandem N-terminal conserved 110 amino acid motifs of 4 left-handed and antiparallel alfa-helices, that forms a hydrophobic pocket able to recognize and bind acetylated lysine residues on histone tails. These PTMs are commonly found in high concentration on active chromatin. By binding to its natural ligand, acetylated lysine, on histones and transcription factors creates a scaffold for transcriptional machinery to come together (Kulikowski et al. 2021).

Under physiological conditions, BET proteins commonly participate in the transcription of housekeeping genes, including elements of the cell cycle, cell identity and apoptosis. At the same time, experimental data have demonstrated the involvement of some BET proteins in many pathological conditions, such as tumorigenesis, infections and inflammation, keeping up the interest on these proteins (Borck et al. 2020).

BRD4 is the most characterized member of the BET protein family and it is known to participate in the transcription of NF-kB-p65-dependent genes. BRD4 participates via interaction with transcription complexes Mediator (Med) and positive transcription elongation factor B (P-TEFb) in the activation and elongation of transcription. P-TEFb is a complex composed of cyclin-dependent kinase 9 (CDK9) and a regulatory subunit, Cyclin T1 or T2. The kinase activity of CDK9 stimulates RNAPol-II activity and elongation by phosphorylation. Two BRD4 regions seem to interact with P-TEFb: the C-terminus domain (CTM) of BRD4 interacts with Cyclin T1 and CDK9, while the BD2 bromodomain interacts with Cyclin T1. These non-histone binding can take place through lysine acetylation-dependent mechanism or not. In particular, the CTM region of BRD4 interacts with Cyclin T1 and CDK9, while the BD2 bromodomain interacts with Cyclin T1 (Fig. 2B) (Hajmirza et al. 2018). Med, a complex that transduces signals from transcription factors and activators at enhancers to promoters, is recruited by BRD4. In conclusion, BET proteins bind to acetylated histones at enhancers, promoters, and transcriptional start site creating a scaffold for the assembly of the transcription machinery facilitating gene expression (Kulikowski et al. 2021). Accordingly, BET proteins, as epigenetic readers, allow the translation of epigenetic marks into transcriptional programs that define the cell state also in relation to prior stimuli. Indeed, the switch- off failure of BET proteins may explain the chronic disease state that persists at the transcriptional level, highlighting their potential role in both metabolic memory and therapeutic application (Borck et al. 2020).

In this regard, numerous studies have shown a fundamental role of BET proteins in promoting chronic condition like atherosclerosis, under persistent and harmful cues (Borck et al. 2020). In response to a variety of mechanical or chemical stimuli, ECs modify their transcriptional program undergoing a reversible cell state transition. These dynamic switches are driven by BET proteins, thus playing a pivotal role in phenotypic plasticity of endothelial cells (ECs). Specifically, Brown et al. demonstrated for the first time that stimulation of ECs by TNFα rapidly deploys NFkB to enhancers and promoters scattered throughout the genome. This causes the creation of new super enhancer regions proximal to canonical genes of inflammatory response in endothelial cells. It was even observed that the BRD4 inhibition through small molecule BET bromodomain inhibitors impedes NF-kB-65-dependent reorganization (Brown et al. 2014).

## Epigenetics and diabetes mellitus

Emerging evidence demonstrates that epigenetics is the way in which the cells remember environmental stressors. Cellular recording of stimuli that can be mitotically inherited represents a biological strategy to facilitate transcriptional responses, e.g. in host immune defenses (Sun and Barreiro 2020). In DM, the interplay between gene and environment not only reflects current glycaemia, but it also mirrors prior hyperglycaemia spikes, an important determinant of micro- and macrovascular complications risk for DM patients (Hanssen et al. 2020; El-Osta et al. 2008).

While the role of persistent epigenetic changes and their association with excessive superoxide (ROS) production in DM metabolic memory is becoming clear (Feng et al. 2013), the role of epigenetics in DM pathogenesis is also now emerging (Ronn et al. 2023). Some environmental factors, like aging, fatty rich diet, and sedentary life, may trigger epigenetic changes that could contribute to T2D onset. Moreover, it has been recently demonstrated that within the islet of Langerhans epigenetic modifications occur and can be detected years before the onset of the disease (Ouni et al. 2020). Other studies suggest a direct link between DNA methylation, gene expression and obesity status: for examples, in obesity condition it was observed a decreased methylation and therefore an increased expression of SOCS3 (Wang et al. 2018), a gene involved in insulin and leptin resistance with severe implication for glucose homeostasis (Pedroso et al. 2019).

## Epigenetic mechanisms in diabetic cardiovascular complications: endothelium disfunction and vascular inflammation

In T2D patients, pro-inflammatory and pro-oxidative pathways correlate with the development of atherosclerosis. Hyperglycaemia mediates persistent chromatin changes in endothelial cells (ECs), and the following studies show a unifying mechanism of damage promoted by oxidative stress in chromatin remodelling and persistent transcription of inflammatory genes, ultimately responsible for progressive damage and dysfunction even after restoration of normal blood glucose levels.

The Src homology collagen (Shc) adaptor protein p66^shc^ is a key regulator of mitochondrial function, ROS production, aging and apoptosis (Galimov 2010; Lone et al. 2018). To date, P66shc is now considered a novel biomarker of oxidative stress and its expression has been found to be upregulated in several types of organ and damaged tissues under diabetic conditions, including in mononuclear cells obtained from T2D patients (Costantino et al. 2018; Mousavi et al. 2022; Biondi et al. 2023).

**Fig. 3:**
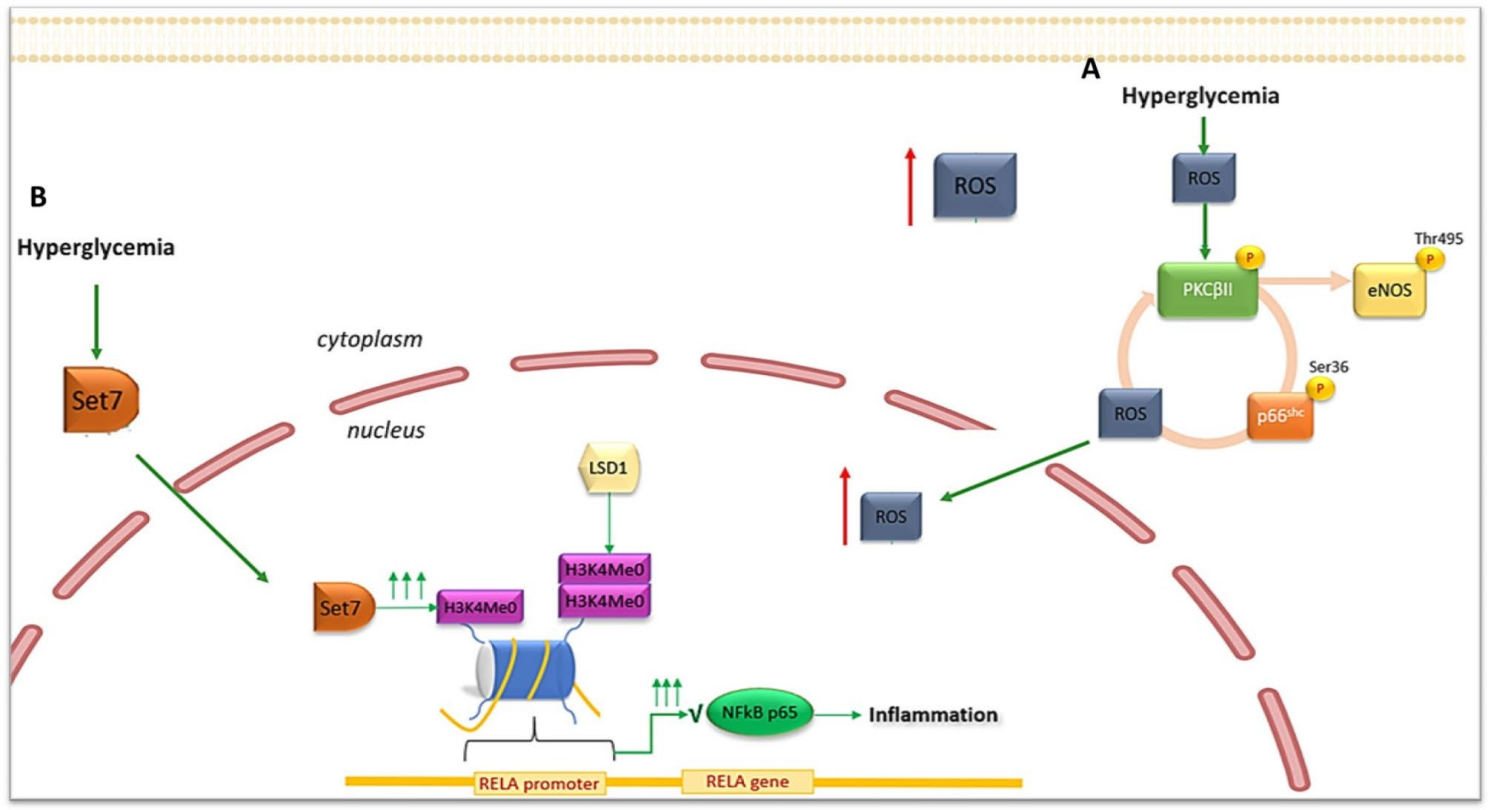
(**A**) Epigenetic-mediated upregulation of the adaptor protein p66shc. (**B**) Set7-mediated regulation of RELA gene transcription

As previously described, oxidative stress triggers the production of DAG and the subsequent activation of PKC. In ECs hyperglycemia-induced PKCβ2 activation promotes both eNOS inhibition by threonine-495 residue (Thr495) phosphorylation (Rask-Madsen and King 2005) and p66shc mitochondrial translocation by serine-36 residue (Ser36) phosphorylation with consequent production of ROS (Paneni et al. 2015a, b) that further contributes to PKCβ2 activation amplifying, in a self-feeding circle, NO depletion, and p66shc and ROS increase. Ultimately, p66^shc^ activation represents both the effect and the cause of ROS production (Costantino et al. 2018; Paneni et al. 2013a, b, c) (Fig. 3A).

Noteworthy, glucose-dependent epigenetic modifications at the level of the p66shc promoter, which persist despite the restoration of normoglycaemia, further contribute to the maintenance of this harmful vicious cycle (Paneni et al. 2013a, b, c). Both DNA demethylation and increased H3 acetylation promoted by the general control nonderepressible acetyltransferase 5 (GCN5) and by the downregulation of the histone deacetylases SIRT1, are responsible for the overexpression and activation of p66shc in diabetic conditions (Padgham 1990). SIRT1 deacetylates H3 histones at the human endothelial p66Shc promoter suppressing ROS production and promoting transcription of SOD, therefore SIRT1 activation could prevent hyperglycemia-induced endothelial dysfunction and avoids hyperglycemic memory (Paneni et al. 2013a, b, c; Zhou et al. 2011).

The activation of p66shc pathway with consequent mitochondrial superoxide production is able in turn to trigger NF-κB-p65 expression and subsequent vascular inflammation (Eelen et al. 2015). In this regard, a great body of evidence elects NF-kB-p65 as the key mediator of vascular inflammation in DM.

In aortic endothelial cells El-Osta and co-workers demonstrated that spikes of hyperglycaemia induced long-lasting epigenetic alteration in terms of H3K4me1enrichement at level of RELA gene encoding for NF-kB-p65 subunit. This modification, causing transcription of NFkB p65 and consequent pro-inflammatory genes expression, persisted up to 6 days after the restoration of normoglycemic conditions (El-Osta et al. 2008). The protagonist in this scenario was the methyltransferase Set7 that under hyperglycaemic condition entered the nucleus causing the mono-methylation of H3K4 (Fig. 3B). Noteworthy, idebenone, a mitochondrial antioxidant molecule, showed to reduce the recruitment of Set7 to RELA promoter (Cooper and El-Osta 2010).

Interestingly, the increased expression of NF-kB-p65 subunit was also sustained by the reduction of di- and tri-methylation on H3 lysine 9 and 14 at promoter level, induced by the lysine-specific demethylase LSD1 (Giacco and Brownlee 2010; El-Osta et al. 2008) and by DNA demethylation at CpGs islands through TET2 upregulation (Scisciola et al. 2020).

Hyperglycemia is the most potent driver of Set7 upregulation and subsequent studies demonstrated a link between Set7-dependent epigenetic modification and vasculopathy in T2D patients. Both Set7 transcript and translated protein were found upregulated in peripheral blood mononuclear cells of T2D patients and correlated with the levels of glycated haemoglobin and fasting plasma glucose (Paneni et al. 2015a, b). Noteworthy, Set7-dependent H3K4 mono-methylation of the NF-kB p65 subunit not only provides a mechanism for epigenetic memory in macrophages but, once transferred to myeloid progeny, also contributes to diabetes-induced trained immunity by increasing the generation of more inflammatory and senescent monocyte subsets from haematopoietic stem cells (HSCs) (Ostuni et al. 2013; Vinci et al. 2024). Targeting this chromatin-modifying enzyme may represent a novel therapeutic approach to prevent atherosclerotic vascular disease in T2D (Paneni et al. 2015a, b). Indeed, gene silencing of Set7 with small interfering RNA (siRNA) in monocytes showed to reduce TNF-α-induced recruitment of NF-kB-p65 to inflammatory gene promoters (Li et al. 2008).

Interestingly, epigenetic abnormalities were also found to be involved in diabetes-induced impairment of the Stromal Derivate Factor 1α / C-X-C chemokine receptor 4 (SDF1α/CXCR4) axis in CD34^+^ HSCs. High glucose exposure of the cells was shown to increase DNA methylation of the CXCR4 promoter, with consequent reduction in gene expression and migratory ability. These changes, which persisted despite the return to normoglycaemic conditions, were also detected and confirmed in HSCs isolated from the bone marrow of T2D patients (Vigorelli et al. 2019).

## Non-coding RNAs and cardiovascular complications of DM

Numerous studies have shown that DM alters the expression of several miRNAs and lncRNAs and their up or down regulation has been implicated in several steps of CV complications in diabetes, including endothelial dysfunction, vascular smooth cell/macrophage phenotype switching, and cardiomyocyte hypertrophy and fibrosis (Prandi et al. 2022). To date, more than 2500 miRNAs have been identified in the human genome, and given their ability to regulate up to 30% of all protein-coding genes, including epigenetic enzyme-coding genes, they are also recognised as modulators of chromatin conformation (e.g. miRNA-34a, miRNA-204 and miRNA-125b). Their pathophysiological significance in DM-induced CV complications is still not fully understood and requires further research (Hanson et al. 2009; Soccio et al. 2022). For additional details on this topic, we refer to dedicated articles and reviews (Prandi et al. 2022; Prattichizzo et al. 2021a, b; Reddy et al. 2014; Tang et al. 2018). The miRNAs, mainly located at the intracellular level, are sorted also extracellularly in various body fluids (Hanson et al. 2009) where they represent a form of intercellular communication. Extracellular miRNAs can evade the activity of RNAs present in biological fluids by being packed into extracellular vesicles (EVs-miRNA) or associated with transport proteins (EVs-free), making them more stable (Soccio et al. 2022). Given their serum stability and reproducibility and consistency among healthy individuals, they can be used as biomarkers in clinical practice for diagnosis, prognosis and response to treatment in many diseases, including cardiovascular complications in T2D (Prattichizzo et al. 2021a, b).

Similarly, lncRNAs have been implicated in various steps of atherosclerosis and DM-associated cardiovascular disease by regulating endothelial cell growth, migration and inflammation (Prandi et al. 2022; Reddy et al. 2014; Tang et al. 2018). Mainly localized into the nucleus where they regulate gene expression, a large fraction of lncRNAs are exported to the cytosol and in the exosomes (Statello et al. 2021). An important family of epidrugs in development includes ncRNA-based therapeutics that are currently in preclinical development, some of which are being evaluated in clinical trials (Poller et al. 2018). Nevertheless, while miRNAs hold great promise for therapy, the studies on lncRNAs are limited because many of them are not conserved between species, making it difficult to translate preclinical results into clinical trials (Prandi et al. 2022) .

## Epigenetic therapies in diabetic cardiovascular complications

Epigenetic mechanisms add an additional layer of complexity to the heterogeneity of cell biology; from one genome, epigenetics create “n” possible epigenome that ensures cells’ identity. Increasing evidence demonstrate that epigenetic aberrations might be a driver of a disease or aggravate its symptoms, conferring epigenetics a central role in both health and disease condition. Whereas genetic mutations are stable and difficult to reverse, epigenetic marks are more easily modulable and reversable: they can be used as emerging tools therapeutic intervention, also for CVD. Several studies demonstrated an altered DNA methylation profile in both atherosclerotic plaque, islets of Langerhans and in T2D human tissues (Avrahami and Kaestner 2019; Zaina et al. 2014; Ling and Ronn 2019). Thus, the pathogenetic pathways underlying the development of T2D and CVD may have an epigenetic origin. For these reasons, the epigenetic modification may represent a target to predict and monitor the T2D development and slow down the onset of diabetes-related complications.

In recent years, a large number of molecules have been developed to affect epigenetic makeup of the genes by modulating the activity of the epigenetic machinery (i.e. writers, erasers and readers). These epigenetic modifiers include histone deacetylase activators (HDACa) histone deacetylase inhibitors (HDACi), sirtuin-activating compounds (STACs), histone acetyltransferase inhibitors (HATi), DNA methyltransferase inhibitors (DNMTi), histone demethylating inhibitors (HDMi), and bromodomain and extra-terminal domain inhibitors (BETi). There are numerous ongoing trials assessing epigenetic drug’s action on a wide range of diseases. However, so far, the use of FDA-approved epigenetic drugs is mostly limited to haematological malignancies, consistent with the increasing epigenetic profiling of cancer: Vorinostat (HDAC inhibitor) for cutaneous T-cell lymphoma, Panobinostat (HDAC inhibitor) for multiple myeloma, and 5-azacytidine and decitabine (DNA-methyltransferase inhibitors) for certain types of leukaemia. In contrast, very few molecules are being investigated for the treatment of CVD in DM, these encompass HDACi, for the treatment of diabetic cardiomyopathy (Garmpi et al. 2022), Set7 inhibitors, to rescue the vascular phenotype (Paneni et al. 2015a, b), and BET inhibitors.

### BET inhibitors compounds

While the other class of epidrugs has been extensively reviewed elsewhere (Costantino et al. 2018; Masi et al. 2021), BET inhibitors (BETi) are emerging as a new epigenetic approach to the treatment of CVD. Epigenetic drugs typically target “writers and erasers”; BETi work by making epigenetic marks that promote atherosclerosis or other cardiovascular diseases unreadable, thus stopping the execution of the epigenetic code. BETi were discovered in 1990s and their application field mainly covers cancer treatment, given their ability to cause G1 cell cycle arrest. Although they remain promising drugs for cancer, especially in combination with other epigenetic modulators, they are now being investigated for the treatment of a wider range of pathological conditions.

The first two BETi were first described in 2010: JQ1 for NUT carcinoma and I-BET 762 for sepsis. They are pan-selective, so they do not discriminate within and across BET family (Neele et al. 2020) (Table 1). JQ1 is a broad-spectrum BET inhibitor, targeting both BD1 and BD2 of all BRDs, and has become the prototypical molecule of its class. It is used in preclinical studies, but not in human clinic because of its short half-life of just one hour. On the contrary, I-BET 762 (Molibresib) shows good potency and pharmacokinetic and is currently in phase II for the treatment of women with HR+/HER2- advanced or metastatic breast cancer (NCT02964507). It has been shown to downregulate the expression of inflammatory genes. I-BET 151 is an optimized form of I-BET 762 and its application is in preclinical studies (Borck et al. 2020).

**Table 1:** List of BETi in preclinical and clinical phase. So far, Apabetalone is the only one in clinical trials for CVD

Drugs	Targets	Stage
Apabetalone (RVX-208)	BD2 domain	Clinical phase 3 for CVD
BI 2536	BRD4	Clinical phase 2
I-BET-762 (Malibresib)	BRD2, BRD3, BRD4, BRDT	Clinical phase 2
CPI-0610	BD1 domain, BRD2, BRD4	Clinical phase 2
INCB054329	BRD2, BRD3, BRD4, BRDT	Clinical phase 1/2
PLX51107	BRD2, BRD3, BRD4, BRDT	Clinical phase 1
AZD5153	BRD4	Clinical phase 1
Birabresib (OTX015)	BRD2, BRD3, BRD4	Clinical phase 1
CPI-203	BRD4	Preclinical
GSK1324726A (I-BET726(	BRD2, BRD3, BRD4	Preclinical
Bromosporine	BRD2, BRD4	Preclinical
BI 894999	BRD4	Preclinical
JQ1	BRD2, BRD3, BRD4, BRDT	Preclinical
I-BET151 (GSK1210151A)	BRD2, BRD3, BRD4	Preclinical
PFI-1 (PF-640S761)	BRD2, BRD4	Preclinical

In addition to pan-BETi molecules, there are a number of BETi compounds that exhibit BD selectivity. Among these, the only BETi in advanced clinical trials stage outside oncology is RVX-208 (apabetalone). Given BET proteins ‘role in the onset and perpetuation of disease state and in inflammation response, BETi are gaining increasing interest as therapeutic agents to attenuate chronic disease progression.

### Apabetalone: in vitro and in vivo results

Apabetalone (RVX-208) is a quinazoline derivative of resveratrol developed by the canadian Resverlogix Corporation, a late-stage clinical biotechnology company. Apabetalone positively affects many biological processes and normalize dysregulated epigenetic mechanism that cause multiple chronic diseases, by inhibiting the action of BET proteins (Borck et al. 2020).

**Fig. 4:**
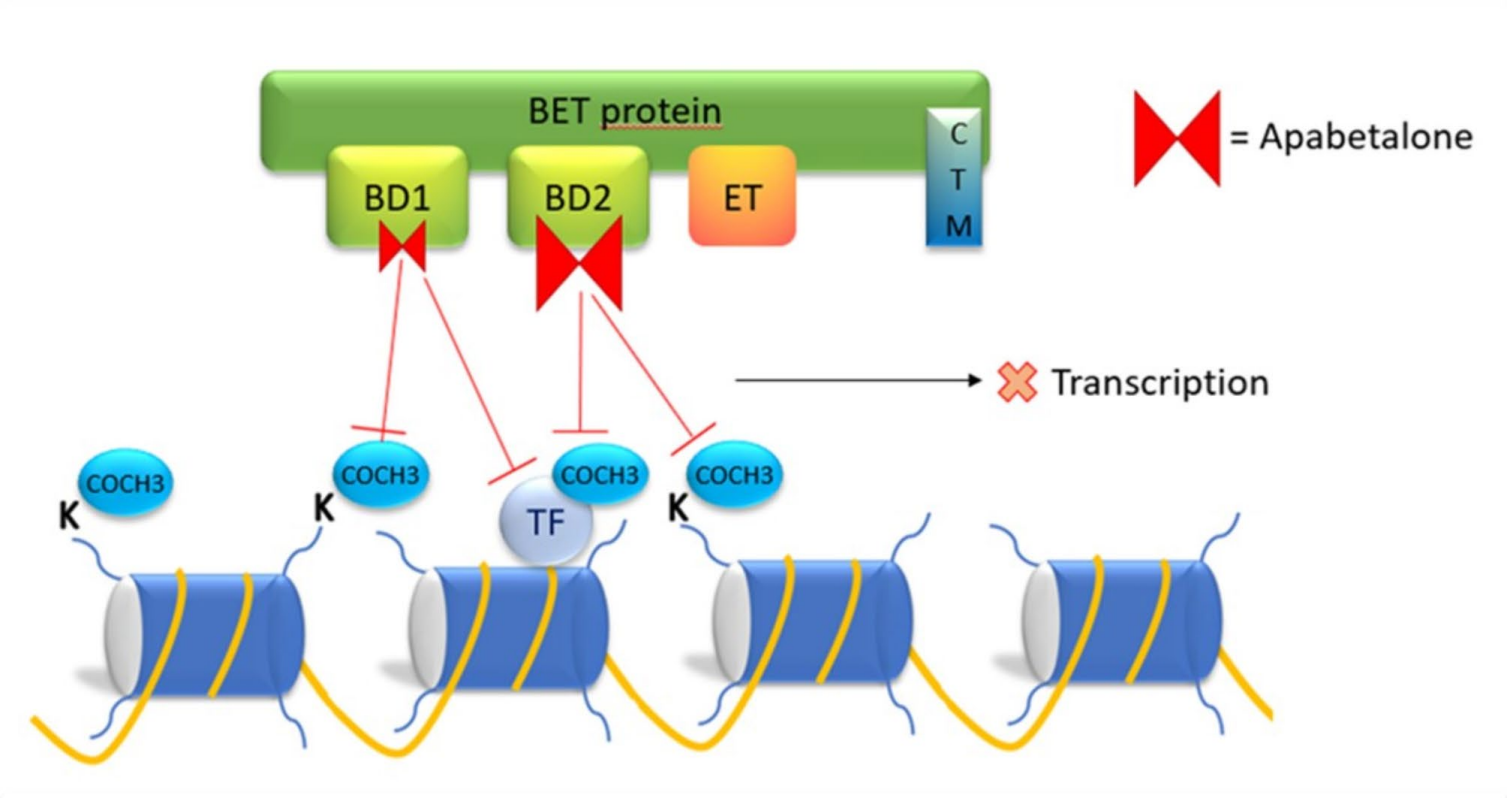
Representative mechanism of action of the BET inhibitor apabetalone

Apabetalone shows a high selectivity for the second bromodomain (BD2) of BRD4. Thus, the drug binds and inhibits BRD4, preventing it from binding acetylated lysine residues on histones and activating gene transcription (Fig. 4) (Gilham et al. 2016). Initially developed as a molecule capable of enhancing the cholesterol efflux capacity of high-density lipoprotein C (HDL-C) from the liver, it has been shown to modulate a wide range of genes involved in inflammation and in the development of cardiovascular disease (Gilham et al. 2016; Bailey et al. 2010).

In ECs, treatment with apabetalone drastically reduces NFkB-dependent cytokines, chemokines and adhesion molecules transcription in response to inflammatory stimuli. The uncoupling of BRD4 from its binding with acetylated RELA, blocks NFkB signalling (Tsujikawa et al. 2019). Tsujikawa and collogues demonstrate that apabetalone reduces the expression of vascular inflammation mediators in vitro and in the plasma of CVD patients as well as the expression of selectins (P-selectin and SELE) in ECs, preventing the recruitment and the early capture on endothelium of monocytes (Tsujikawa et al. 2019). Notably, Mohammed et al. demonstrated in a mouse model of diabetes that apabetalone is able to prevent maladaptive transcriptional programmed in ECs, thereby preserving angiogenic properties and post-ischemic vascularization after hindlimb ischemia (Mohammed et al. 2022).

Monocytes isolated from T2D and CVD patients showed a higher baseline levels of cytokine gene expression (IL-1α, IL-1β and IL-8) compared to control monocytes and a hyper-responsiveness to inflammatory stimuli ex vivo. In response to stimulation with interferon γ (IFNγ), the NFkB pathways was upregulated of 30% compared to control monocytes. Ex vivo apabetalone treatment of the cells showed to abolished pro-inflammatory hyper-activation of T2D monocytes by reducing TLR and cytokine gene signature (Wasiak et al. 2020). In addition, the same authors demonstrated the ability of the novel molecule to downregulate complement cascade in vitro, in mice and also in patients with CVD (Wasiak et al. 2017).

### Apabetalone reduces the serum level of alkaline phosphatase and the incidence of major adverse cardiovascular events: the BETonMACE trial

A post-hoc analysis of pooled data from the ASSERT, ASSURE and SUSTAIN trials showed that apabetalone lowered in a dose dependent fashion serum alkaline phosphatase (ALP), a modifiable cardiovascular risk marker, and reduced the incidence of MACE. Interestingly, the magnitude of benefit appeared to be correlated with the extent of ALP reduction.

This led to the phase III BETonMACE clinical trial, a randomised, double-blind, placebo-controlled study in which the effects of apabetalone on MACE and hospitalizations were compared with placebo (Kalantar-Zadeh et al. 2021). The first phase III clinical trial of an epidrug in the treatment of diabetic CV complications and outside the field of oncology.

The BETonMACE trial (NCT02586155) was conducted in 13 countries and recruited 2425 patients with recent (7–90 days) acute coronary syndrome (ACS), T2D and low HDL (≤ 40 mg/dl for men, ≤ 45 mg/dl for women), including 288 participants with chronic kidney disease (CKD). The patients enrolled were treated orally with apabetalone 100 mg/12 h or placebo for 120 weeks. The primary composite outcome was the time to first occurrence of cardiovascular death, non-fatal myocardial infarction, or stroke. The apabetalone was generally well tolerated with an overall incidence of adverse events similar to the placebo group. However, more patients allocated to apabetalone than placebo discontinued the trial because of elevations of liver enzyme levels. The study showed that after a median follow-up of 26.5 months, there were 274 primary endpoints, 125 (10.3%) in the tested-drug group and 149 (12.4%) in the placebo group, with an estimated event rate of 7.8% in the apabetalone group and 9.7% in the placebo group at 18 months (Kaplan-Meier curve), but the difference was not statistically significant, therefore the study concluded that apabetalone did not reduce cardiovascular events compared to placebo (Ray et al. 2020). However, secondary endpoint analysis showed a nominal significance for decreased rate of hospitalization for congestive heart failure in the treatment group in comparison to placebo (HR = 0.59 for first hospitalization for congestive heart failure and HR = 0.49 for first and recurrent hospitalization) (Neele et al. 2020; Ray et al. 2020). An important limitation of the study was that it was underpowered to detect small differences between groups, due to lower than expected event rates and the fact that the study population was based on an 80% power to detect a 30% reduction in events, which was not achieved. Different results could therefore be obtained with a larger cohort. However, additional subgroup data analysis suggested that apabetalone had a greater effect on the primary outcomes in patients with impaired renal function or low LDL-c levels (Neele et al. 2020; Ray et al. 2020). Moreover, the trial also showed that among T2D patients, those with CKD were highly responsive to apabetalone treatment, with a 50% nominal reduction of MACE over 27 months (Kalantar-Zadeh et al. 2021).

Concluding, it is not time yet to use apabetalone in patients; nevertheless, even though there was no statistical significance among primary outcome and many of the second endpoint, the trend was consistent and encouraging. An adequately powered and larger clinical trial is necessary to carefully value the effect of apabetalone on MACE events. In confirmation of this, on February 2020 Resverlogix received the FDA Breakthrough Therapy Designation for apabetalone in combination with top standard care “for the second prevention of major adverse cardiac events in patients with type 2 diabetes mellitus and recent acute coronary syndrome”. This important recognition will facilitate the programming of future clinical trials and a time-efficient drug development.

### Antidiabetic drugs with epigenetic activity

The antidiabetic drugs currently available for the treatment of T2D can control blood glucose levels by different mechanisms. Some of them have been shown to have beneficial cardiovascular effects in addition to their glucose-lowering action, which may be related to their ability to inhibit diabetes-induced epigenetic changes. Among these, metformin has for example shown to reduce cardiovascular disease in diabetics. This biguanide compound reduces hyperglycemia by increasing insulin sensitivity and decreasing hepatic gluconeogenesis. Its beneficial effect at cardiovascular level seems to be in part due to the restoration of optimal glucose concentration and metabolism, and also to pleiotropic epigenetic mechanisms that include the ability of regulating ncRNAs expression and DNA methylation (Yang et al. 2022; Bridgeman et al. 2018; Wang et al. 2020; Elbere et al. 2018). By regulation of miRNAs expression metformin has shown to ameliorate insulin resistance and prevent diabetic complications such as nephropathy, retinopathy and cardiomyopathy. In addition, through the activation of its main signalling pathway, AMPK, metformin has shown to affect the epigenetic makeup of the genes and their transcription by modulation of epigenetic enzymes activity (e.g. DNMTs and HATs) (Bridgeman et al. 2018; Giordo et al. 2023).

Furthermore, epigenetic mechanisms also seem to participate in the cardiovascular protective effects of GLP-1 receptor agonists (GLP-1RAs) and sodium-glucose cotransporter-2 inhibitors (SGLT2i), a novel class of antidiabetic drugs (Serowik and Pantalone 2024). Although both groups of drugs have beneficial effects, they have slightly different profiles. The SGLT2 inhibitors have shown a better effect in terms of a reduced incidence of HF, while the GLP-1-RAs have shown a reduced risk of CV events, in particular stroke (Rolek et al. 2023). GLP-1RAs showed in human aortic endothelial cells exposed to high glucose and in diabetic patients to prevent glucose-induced DNA demethylation of NF-kB promoter by reducing TET2 binding. This results in reduced NF-kB-65expression and activation of inflammatory target genes (IL6 and TNFα) with beneficial effects on vascular inflammation (Scisciola et al. 2020). Similarly, the efficacy of SGLT2i in improving cardiac function involves epigenetic mechanisms. Dapaglifozin has been shown to upregulate miRNA-30e-5p, an ncRNA that reduces cardiomyocyte autophagy, and the expression of miRNA-199a-3p. The latter, by decreasing cardiac PPARγ levels, restores mitochondrial oxidation of fatty acids and improves cardiac function in patients with HF (Solini et al. 2019). Dapaglifozin has also been shown to exert a nephroprotective action, preserving renal vasodilation by downregulating miRNA-27b (Solini et al. 2019).

## From epigenome to personalized therapy

Epigenetic therapy is a challenging area of research and development. Epigenome analysis between diseased and healthy individuals can provide information about the epigenetic changes involved in disease. However, it requires a large number of samples and the target epigenetic markers must be stable over time. In addition, while the epigenetic profile is easy to assess in circulating cells, it is not the same for all cells of the tissues, which are characterised by a heterocellular composition. The epigenome also depends on a wide range of environmental, physiological, social and behavioural variables, unlike genetic variants. It is therefore difficult to assess the effect of all these factors on a specific DNA methylation or PTM at the level of a single cell and the effect of such cellular changes at the tissue level (Santalo and Berdasco 2022). Thus, variability, together with the reversibility through the life course of the epigenetic landscape, pose a lot of question whether epigenetic variations are to consider cause of the disease or merely a consequence of it (Santalo and Berdasco 2022; Martin et al. 2011). Longitudinal intervention studies can determine whether the epigenetic signature is a result of exposure to the disease environment; however, it remains unclear whether epigenetic changes may actually represent a target for in vivo treatment or whether they represent an epiphenomenon of a broader ‘response’ of cells to exposure, in which epigenetic signs may play only a marginal role (Martinez-Moreno et al. 2020).

Next, epigenetic marks are highly dynamic, complex and spatio-temporal specific, and this requires epidrugs to be equally specific, otherwise the treatment would lead to a range of side-effects which represent one of the main challenges of epigenetic therapies. Epigenetic reprogramming to modulate gene expression modifications using selective small molecule like apabetalone or SET7 inhibitors such as (R)-PFI-2 might mitigate limitations.

Large-scale epigenomic sequencing is an excellent tool for generating cell-specific epigenetic maps. Several projects are underway, including the Epigenome-wide Association Study (EWAS) and the Human Epigenome Project (HEP). Their aim is to provide a high-resolution reference human epigenome maps both for health and disease cell-types that can be exploited for diagnosis, drug optimization and drug discovery. This would be useful to derive association between a cell-specific epigenetic signature and a phenotype or transcriptional output (Gjaltema and Rots 2020).

Moreover, given the high diversity of chromatin structure among patients, the ultimate and most challenging goal is to transform current standard therapies into personalised epigenetic therapies. The novel epigenetic editing techniques seem to have the answer (Gjaltema and Rots 2020). This novel approach is based on locus-specific targeting of the genes to modify their transcription. It consists on DNA binding platform derived from zinc finger nucleases (ZFNs), TALEN and the more precise and just awarded CRISPR/Cas9, fused to epigenetic modifiers in order to target specific DNA sequences (Ueda et al. 2023). When considering both gene or epigenetic therapies, safety is crucial; since epigenome editing does not cause double-strand DNA breaks, it is safer and the off-target effects are mostly silent (Mlambo et al. 2018). However, the remote effects of epigenetic modifications on the chromatin landscape should be considered. Given the close interaction between epigenetic marks and chromatin-binding proteins, it is important to consider that the intervention on one factor may induce unexpected effects on other components of the complex. Thus, although still in its infancy, epigenome editing could be a powerful tool in the future (Mlambo et al. 2018).

## Conclusion

Although tight glycaemic control is undoubtedly the best option for preventing or slowing the progression of diabetic complications, some patients may find difficult to achieve lifelong control. Today, increased knowledge of epigenetic mechanisms is opening up new perspectives in the diagnosis, prognosis and therapy of cardiovascular disease. A better understanding of the fine-tuning of epigenetic mechanisms at work in diabetic complications, and the identification of epigenetic biomarkers for early detection of the onset and progression of diabetic complications, promise to drive an unprecedented advance in precision medicine.

## Data Availability

No datasets were generated or analysed during the current study.
